# Molecular, histological and microcirculatory modeling of cerebral ischemia in pigs

**DOI:** 10.1186/cc10911

**Published:** 2012-03-20

**Authors:** O Suchadolskiene, A Pranckunas, B Kumpaitiene, P Dobozinskas, Z Dambrauskas, V Veikutis, K Stasaitis, G Baliutyte, D Vaitkaitis, V Borutaite

**Affiliations:** 1 Lithuanian University of Health Sciences, Kaunas, Lithuania; 2 Institute of Cardiology, Lithuanian University of Health Sciences, Kaunas, Lithuania; 3 Institute of Neurosciences, Lithuanian University of Health Sciences, Kaunas, Lithuania

## Introduction

Ischemic brain injury due to stroke and/or cardiac arrest is a major health issue in modern society requiring urgent development of new effective therapies. The use of appropriate animal models is essential to study the mechanisms of ischemia-induced injury and neuroprotection. The goal of our study was to establish a reliable and reproducible model of brain ischemia in pigs (with the ischemia-induced microcirculatory, mitochondrial and structural alterations) for further research.

## Methods

Eighteen pigs (18 to 22 kg) were anesthetized and randomly assigned to the one of the following groups: 1 - control, 2 - unilateral carotid occlusion, 3 - bilateral carotid occlusion, 4 - bilateral carotid occlusion + hypotension (MAP 40 to 50 mmHg). In order to investigate the effects and mechanisms of cerebral ischemia, we assessed the mitochondrial respiration (high-resolution respirometry), microcirculation (*in vivo *SDF videomicroscopy) and histological structure (light microscopy) of brain tissue in healthy control animals and after 3 hours of brain ischemia (three different models).

## Results

LEAK respiration (measured in the presence of pyruvate + malate but without ADP) was not affected by ischemia in any model. The OXPHOS capacity with pyruvate + malate as substrates decreased by 20% and 79% compared to the control level after bilateral carotid artery occlusion and bilateral carotid occlusion + hypotension, respectively, resulting in the decrease of RCI (ADP/PM) by 14% and 73%. The OXPHOS capacity with succinate as substrate remained constant after unilateral carotid artery occlusion but decreased by 53% after bilateral carotid artery occlusion and hypotension compared to the control level (*P *< 0.05, *n *= 3 to 6). Mitochondrial respiration rates after addition of atractyloside and cytochrome c were the same in all experimental groups, suggesting that intactness of mitochondrial outer membrane was not affected by cerebral ischemia. Microcirculatory and histological alterations also demonstrated increasing derangement and reversible structural changes after bilateral carotid occlusion and vascular occlusion combined with systemic hypotension.

## Conclusion

The experimental model of bilateral carotid artery occlusion and systemic hypotension-induced cerebral ischemia in pigs is a useful tool to investigate the mechanism of cerebral ischemia and/or neuroprotection (medications, hypothermia, and so forth).

